# Defending the Integrity Principle: Necessity, Remorse and Moral Consistency in the Protest Trial

**DOI:** 10.1093/ojls/gqaf003

**Published:** 2025-03-14

**Authors:** Steven Cammiss, Graeme Hayes, Brian Doherty

**Keywords:** protest, trials, remorse, necessity, duress

## Abstract

The protest trial has distinctive features and should be governed by what we term the ‘integrity principle’: it should respect the moral consistency of the defendant; justifications, not excuses, should be privileged; and the ‘remorse principle’ should not apply. As such, the trial should enable effective communication where the defendant is held to account in meaningful terms. We apply this argument to three high-profile protest trials: the Frack Free Three; the Stansted 15; and the Colston 4. Using observation data, we argue the first two trials and subsequent appellant court rulings failed to respect the integrity principle. The third case provides a contrast: the defendants maintained moral consistency, and gave an authentic and contextualised account. This was, however, at some cost of political divestment. Nevertheless, the Colston 4 trial is exceptional in a process that typically pays little operational respect to the integrity principle.

## 1. Introduction

The criminal trial, while focused on an epistemic search for truth, is more than an exercise in adjudication; non-epistemic concerns are also important.^1^ We think that this is central for (what we will for brevity’s sake henceforth call) protest trials;^2^ we will provide what we believe to be the first conceptualisation of the protest trial, identifying its distinctive features and developing what we term ‘the integrity principle’ (which we will outline shortly). Core to the integrity principle is an understanding of the trial as a *communicative endeavour*; Duff, in asserting a normative framework for the trial, notes it is a search for truth, and as such is also a communicative enterprise that *should* seek to hold the defendant to account for their actions through the attribution of *responsibility*.^3^ However, responsibility is a necessary, but not sufficient, condition for finding liability; a defendant may offer a legally recognised *account* of wrongdoing that amounts to either a justification or an excuse. Such ‘remedial work’ operates, according to Goffman, in a manner that changes the meaning of our acts, thereby rendering action ‘acceptable’.^4^ As protesters claim to be acting *as* citizens (and, therefore, acting on behalf of the community), we argue that the attribution of responsibility and the remedial work that defendants perform in the trial should be different in the protest trial when compared to ‘ordinary’ trials. Drawing on legal, normative and sociological examinations of responsibility, accounts and remorse, we will set out the ‘integrity principle’ that *should* govern the terms of the protest trial, that enables protesters to account for their actions with moral consistency. However, we show how current trials of protesters fall short of this ideal, using three trials to illustrate this.

The criminal trials of non-violent direct action protesters (henceforth ‘direct action protesters’) have very different features from ‘ordinary’ criminal trials. As we shall see, this is a claim dependent on contrasting understandings of the role of justification in the communicative regimes of protest trials and ordinary trials. Ordinary trials do not, except in cases of self-defence and similar defences, centre on the justification of the alleged offence; rather, they focus on the demonstration of guilt. In these trials, the expression of remorse by a defendant is considered central to the law’s ‘truth production’ and moral reordering vocations.^5^ Indeed, the tightly bound dynamics of confession and contrition are widely seen as forming a vital bridge from the acceptance of guilt to the ‘ethical transformation’ of the self, which is considered to be at the heart of rehabilitation.^6^ This transformative aspect is key: both Proeve and Tudor^7^ and Weisman^8^ define remorse as a dynamic moral process, moving through phases of acknowledgement, internal strife and self-transformation. For Weisman, judicial authority brings about a ‘separation between act and being’, as the defendant must demonstrate, through remorse, that ‘the self that is loyal to community is more real than the self that has betrayed community’.^9^ Despite, as Bandes^10^ underlines, an absence both of a clear understanding of what constitutes a display of remorse and of evidence of its relationship to reoffending, these broad principles guide sentencing decisions. In England and Wales, as elsewhere, previous relevant convictions are regarded as aggravating factors, while positive good character and remorse are seen as mitigating factors.^11^ Remorseful offenders can typically expect reduced censure, ‘in light of the offender’s understanding and rejection of their wrongful conduct’.^12^ The relationship of remorse to mitigation is so well established that Tudor refers to it as the ‘remorse principle’.^13^

In protest trials, however, confession and contrition should not *a priori* play this role in either prosecution or sentencing processes, for reasons linked to the possibilities of the protest trial as a continuation of political struggle and a site of citizenship. In contrast to the remorse principle at the heart of ordinary trials, we consider protest trials, for this reason, to be more appropriately governed by what we call the ‘integrity principle’. This principle consists of three elements. First, remorse is not appropriate as protest defendants do not usually dispute the facts of their action/offence but, where able, argue their actions were justified,^14^ as ‘a matter of self-respect and moral consistency’.^15^ For direct action protesters, a defence of necessity is an attractive option, because it ‘allows the public-spirited lawbreaker to deny guilt without denouncing his politically motivated acts’,^16^ in a manner that respects their autonomy.^17^ Second, protest defendants may be expected to deny that they have betrayed the community but, rather, argue that they have acted in the community’s collective name for its moral benefit. As remorse is an expression of moral regret, it would, as Bennett notes, be ‘grotesque to expect someone to display remorse when they are not morally guilty’;^18^ indeed, for Brownlee, those committing acts of civil disobedience are attempting ‘to engage society in a dialogue’ about justice and have ‘good reason not to want to recite the imposed, generic script of the … apology ritual of punishment’.^19^ Third, UK courts should respond in a manner that respects protest defendants’ philosophical beliefs.^20^ Lord Hoffmann in *Jones* recognised the importance of this by observing that the sincerity of a protester’s commitment (and thus their *absence* of remorse) may be considered a *mitigating* factor.^21^ Such cases therefore involve a ‘special category’ of defendants, where leniency may be applied in a way that is contrary to normal sentencing principles;^22^ here, no ‘separation of act and being’ (to adopt Weisman’s terms) is required for the court to show leniency. These three elements of the integrity principle are linked, and they highlight the importance of protest defendants being able to express themselves with integrity and allow them to display moral consistency. The principle reflects that protesters act in good conscience for the common good. The integrity principle, therefore, applies to both the operation of trials and the sentencing of protest defendants.^23^ As will be clear from our analysis, we are calling for a reform to the law to reflect the integrity principle in these cases, through developments of legal defences and sentencing principles.^24^

The integrity principle therefore expresses what we believe should be the different normative understanding of justification in the trial of direct action protesters when compared to an ordinary trial. The centrality of integrity in such cases means the separation of act and being typical of an ordinary trial should not apply. Protest defendants may accordingly be expected to accept, explain and justify their actions in *law*, and seek to persuade the court of their moral rightness, rather than deny them or seek forgiveness for their wrongness. As we shall argue here, evidence from three recent prominent trials of direct action protesters in the English and Welsh courts suggests that this principle has little (and decreasing) purchase; that, in practice, the courts effectively fail to make this distinction between protest and ordinary trials; and that this weakens protest rights.

In this article, we discuss three prosecutions: the conviction for public nuisance, imprisonment and release on appeal of three anti-fracking activists, known as the ‘Frack Free Three’, in 2018; the conviction of 15 anti-deportation activists, known as the ‘Stansted 15’, on terrorism-related charges, and the subsequent overturning of this conviction in 2019 by the Court of Appeal; and the trial in 2021–22 of four activists prosecuted for criminal damage for their various roles in pulling down the statue of slaver Edward Colston and dumping it in Bristol harbour. The first two cases ultimately resulted in lenient sentencing: the Frack Free Three’s sentences were overturned on appeal, their prison terms replaced by conditional discharges; and the Stansted 15, faced with possible sentences of life imprisonment, were sentenced to community service orders and suspended sentences, before their convictions were overturned.^25^ Meanwhile, the trial of the ‘Colston 4’ ended with the jury’s acquittal of the defendants.

At first sight, therefore, the discounted sentences ultimately imposed in the first case, imposed and then overturned in the second case, and the acquittal in the third case appear to conform to the protection of the defendant’s philosophical beliefs, where sincerity of commitment trumps the ‘ordinary’ requirements of fault and remorse. Yet, close attention to each of these cases reveals a much more troubling picture. Through observation of the Stansted 15 and Colston 4 trials and the Frack Free Three appeal hearing, and discussion of charging practices, legal defences and judicial decision making, we find that remorse played a pivotal role in the Frack Free Three’s release; the judicial management of the Stansted 15 trial forced a ‘separation of act and being’ on the defendants, severely limiting their capacity to give an adequate account of their action; and the Crown Prosecution Service’s (CPS) decision to bring severe (indeed, excessive) charges in each case was central to the production of these dynamics. Meanwhile, although the Colston 4 case looks much more like the type of trial we would expect from our distinction between protest and ordinary trials—the defendants were able to set out at length the reasons for their action—even in this trial, they did so by carefully presenting themselves as *not activists*, implicitly recognising the figure of the protester as illegitimate. In all three cases, therefore, defendants undertook or were required to undertake a form of separation of act and being, in a way that is inconsistent with the integrity principle that we argue ought to operate in protest trials. As a result, although the *outcomes* of all three cases appear consistent with the protection of protest rights, the *terms* and process by which the outcomes were achieved were much less positive.

In this article, we therefore provide a conceptualisation of the protest trial as a distinct form of trial, identifying its unique features and the central function of the integrity principle. We argue that the separation of act and being, identified by Weisman to be central to the operation of ordinary trials, should not operate in protest trials, because in these trials act and being should not be separated. We develop this argument in the first part of the article, bringing understandings from sociological literature concerning responsibility and remedial work into dialogue with normative legal analysis, distinguishing between apologies and accounts, justifications and excuses, duress and necessity. We summarise the three cases and discuss our methods, including our ethnographic approach to observing these trials. We then examine the Court of Appeal’s ruling in the Frack Free Three case, the terms of the Stansted 15 defence and how the Colston 4 trial offers a glimpse of a different way of proceeding. We conclude that, due to constraints imposed by the court in each case, the Frack Free Three hearing’s emphasis on remorse, the effective disarming of justification in the Stansted 15 trial and the Colston 4’s disavowal of the label ‘activist’ create worrying precedent for the protection of protest rights. The significance of our argument lies, therefore, not just in its conceptual ambition, but also in the case we make for why the protest trial should be understood as distinctive.

## 2. Accounting for Action: Responsibility and Justification in Protest Trials

The distinction we make between ordinary and protest trials necessarily centres on how we understand the *purpose* of a trial. As noted above, writers on the criminal justice process recognise that the criminal trial (of any kind) raises both epistemic and non-epistemic concerns; while the primary focus of the trial is truth finding, other values are equally important.^26^ Duff makes an important contribution here, linking epistemic values to a conception of the trial as a *communicative endeavour*. Truth is a ‘positive goal’ in itself, but one that seeks to hold the defendant *responsible* by calling them ‘to answer to a charge of wrongdoing’ in a way that respects their agency.^27^ A trial that does not respect the defendant’s agency, excluding their participation, consequently ‘becomes a travesty’.^28^ Here, agency is not just a question of autonomy, but one of responsibility; for Duff, criminal responsibility is analogous to moral responsibility, and both are relational; individuals are responsible *to* others *for* their actions.^29^ But, for Duff, criminal responsibility is not merely dyadic (responsibility *for* an action *to* another), but is triadic; people are responsible *as* citizens (and members of a moral community), *to* that community, *for* any public wrongs that they commit. Responsibility, therefore, is framed by the context and communities in and for which action is undertaken, and those who demand that individuals account for their actions must have ‘standing’ to do so.^30^

Furthermore, the requirement that individuals be held to account as citizens means that the moral wrong *for* which they are being held responsible ‘must address [them] in terms of the values that supposedly structure [the] polity’.^31^ The obligations citizens owe each other *qua* citizens is a reflection of how they ‘are related to each other, to the state and to the laws that bind [them]’.^32^ Moreover, the criminal law is not an imposition from a sovereign, but is instead a ‘“common” law’ that reflects the ‘values that [they] share as members of a political community’.^33^ This assertion is important for the protest trial, where, as noted above, defendants often do not deny that their actions breached the law (they accept responsibility) but, rather, provide an account that justifies their actions; they seek, in other words, to claim that their actions are undertaken on behalf of the community, to protect or espouse collective values. In so doing, protesters are challenging the injustice of the prevailing political and social arrangements; as such, they are acting *as* citizens.

Importantly, Duff recognises that the trial can provide an opportunity to ‘put the law, and the polity whose law it is, on display, and thus opens the way to critical reflection’.^34^ Protesters’ actions, as political, due to their aims and motivations, and the challenge they offer to the state, can lead to questions as to the suitability of judgment; holding protesters to account *as* citizens should accordingly be done ‘with suitably clean collective hands and with clear consciences’.^35^ The direct action protester often suggests that the community does not have ‘clean hands’, having failed to address an ongoing and serious injustice.

The final point to make here is that responsibility is a necessary, but not sufficient, criterion for liability: defendants can avoid liability if they are able to offer a legally recognised account of their actions. In other words, defendants can answer for their wrongdoing by offering a justification that denies their actions are wrongful, or an excuse that admits wrongdoing but provides reasons to avoid liability. This question of accounting for action is therefore central to the protest trial, and has significant implications for questions of remorse, duress and necessity, or what we might call the type of ‘remedial work’ that the trial is structured to address.

### A. Remedial Work: Remorse, Duress and Necessity

In his micro-sociological discussion of public norms and behaviours, Goffman refers to ‘remedial work’ as ‘the provision of corrective readings calculated to show that a possible offender actually had a right relationship to the rules’.^36^ For Goffman, the accomplishment of remedial work in everyday situations enables leniency to be shown for a transgression, and for the transgressor to be subsequently reintegrated into the moral community. To do this, remedial work changes the meaning of the act, ‘transforming what could be seen as offensive into what can be seen as acceptable’.^37^ Two broad strategies of remedial work are available: *accounts* and *apologies*. Where norm-breakers give ‘accounts’, they seek to redefine the situation by means of explanation or justification, whether distancing themselves from the infringement or by arguing the infringement does not in fact violate collective norms and constitutes a reasonable course of action in the circumstances. Where accounts are not available, however, norm violators can develop sanction reduction strategies through expressions of remorse and regret, re-establishing their stable social identity as non-transgressive and that they are ‘worthy of being brought back into the fold’.^38^ As Tavuchis points out, this structure is relational: an apology is a ‘special kind of enacted story whose remedial potential, unlike that of an account, stems from the acceptance by the aggrieved party of an admission of iniquity’, and is dependent on the perceived authenticity of the act.^39^

This is consequential for understanding the difference between ordinary and protest trials. Remorse, it can be seen, is a form of *apology*, an admission of moral guilt; here, remedial work is required to delineate, and subsequently make good, the separation of act and being. In contrast, in protest trials, there is no such separation; remedial work can instead be expected to take the form of an account arguing that collective norms have not, in fact, been violated. Here, the necessity defence acquires specific significance: it is an *account*; necessity *justifies* norm breaking, where, in the reasonable belief of the transgressor, it is a proportionate act committed to prevent a greater imminent harm. For direct action protesters, a necessity defence offers two advantages: a basis for developing moral and political arguments that underpin their action;^40^ and the opportunity to challenge, define and communicate a given public (or corporate) policy as harmful, entailing a ‘collective, normative statement about what should be done in response to individual or social distress’.^41^ For Martin, necessity has radical transformative potential: it affords citizens a legal and democratic mechanism to circumvent political failures and challenge the abuse of power, even where these failures or abuse are legally authorised. Necessity, in theory, is a ‘direct, doctrinally legitimate, and uniquely powerful means through which revolutionary change might be accomplished within prevailing jurisprudential and constitutional structures’,^42^ inherently privileging the provision of collective goods and the alleviation of social distress over the maintenance of private property rights and individual interest.^43^

Excuses, however, do not share this transformative potential to the same extent. While they share elements of Goffman’s definition of accounts, in that they provide reasons for action, they also share elements of his definition of apologies. When offering an excuse, it is accepted that the action was wrong—to adopt Tavuchis’s term, there is an admission of ‘iniquity’^44^—but there is an appeal to forgiveness due to the circumstances of the wrongdoing.

Unsurprisingly, attempts to plead necessity in protest trials are simultaneously increasingly common and subjected to judicial control. In the United States, courts have severely restricted the grounds on which necessity may be pleaded in protest cases.^45^ Nonetheless, attempts to plead necessity in climate change trials, spurred by the 2009 trial of Tim DeChristopher, have proliferated.^46^ However, widespread refusal to put the defence to a jury leads Rausch to note that success is more accurately measured by being able to make the case to a jury, rather than of an acquittal.^47^ In England and Wales, necessity defences are broadly rejected by the courts in protest cases. Where charged under the Criminal Damage Act (CDA) 1971, protesters are able to plead the offence-specific defence of lawful excuse. Juries have frequently acquitted protesters where this is available, such as at Liverpool Crown Court in 1996,^48^ Maidstone Crown Court in 2008^49^ and in a series of trials involving activists who had destroyed genetically modified crops.^50^ In contrast, the House of Lords stated in *Jones*^51^ that the general defence of necessity was *not* available in protest cases.^52^ In *Jones*, Lord Hoffmann set the terms of what we call ‘Hoffmann’s bargain’: protesters who ‘behave with a sense of proportion’ can expect the state to ‘behave with restraint’ in sentencing and charging decisions, but they must ‘vouch the sincerity of their beliefs by accepting the penalties imposed by the law’.^53^

In England and Wales, therefore, protesters may wish to justify their action through a necessity claim; yet the defences available are effectively only of lawful excuse^54^ or duress of circumstances.^55^ Whether the plea of duress may be put to the jury is, further, subject to the gatekeeping power of the trial judge. Given the lack of detailed analysis of the trials of direct action protesters, we know little about how these distinctions and prohibitions work in practice. Forensic ethnography is predominantly preoccupied with the behavioural observation of legal professionals and court systems,^56^ whilst discussions of the English courts are concerned with ordinary trials.^57^ In contrast, discussion of the ‘extraordinary’ prosecutions of direct action protesters overwhelmingly concerns US case law.

### B. Duress, Necessity and Mitigation

We consider this gap in the literature to be consequential: we may *a priori* consider the duress and necessity distinction to be significant to the trial’s functioning, because it affects the claims that direct action protesters offer in their defence. Necessity is a defence ‘involving a choice between two evils’, where there is no ‘wrong where the lesser evil is selected’,^58^ whereas duress is a response to ‘a threat or danger of death or serious injury’ which is ‘imminent’.^59^ In both England and Wales and the United States, the distinction between duress and necessity is commonly blurred, and the two are often, in practice, conflated;^60^ the CPS guidelines, for example, note that the law tends to treat duress of circumstances and necessity as ‘one and the same’, and set the crucial test to be ‘not whether the defendant’s actions were justified, but whether he can be excused on the grounds that a reasonable person would have acted in the same way’.^61^ However, each has different structural and symbolic properties: whilst some forms of necessity are arguably justificatory (an account), duress is *excusatory* (which, as explained above, has elements of an apology).^62^ For Fletcher,^63^ this distinction is important: justification ‘renders conduct right’,^64^ conferring an entitlement to engage in the given act; whereas ‘by definition the conduct that is arguably excused constitutes a wrong’, and is ‘an unjustified violation of a criminal prohibition’.^65^ In other words, whilst ‘a justification negates the social harm of an offense, an excuse negates the moral blameworthiness of the actor for causing the harm’.^66^

In practice, we may expect a duress-based defence to centre less on the balance of harms (and thus of moral assessments over competing values and norms) than on the imminence of harm (and thus over the narrower terms of immediate threats).^67^ This matters: in a case of direct action protest, to mount an excusatory defence is to accept that the act committed was, under normal conditions, wrong. It is, therefore, politically divesting, a form of remedial work that explains action as a consequence of compulsion; it is a separation of *political* act and *moral* being. Analytically, therefore, despite their blurring, duress is sufficiently different to necessity; indeed, it has more in common with an expression of remorse. While excusatory claims may relate ‘conduct to grounds of action that go beyond the simple individual decision to do something’,^68^ as we shall see, they do so in ways that limit the extent to which defendants can account for their actions with integrity. In such circumstances, we should ask: what a claim of duress allows and how it is structured in the situational context of the trial; how it practically affects the presentation of a defence at trial by direct action protesters; how it operates to allow defendants to account for their actions so that the trial is an effective communicative endeavour; and in what sense this is commensurate—or not—with our claim that the process should protect the expression of philosophical belief within the protest trial.

In addition to addressing justifications (necessity) and excuses (duress), we also want to touch briefly upon a third category, mitigation. This is similar to an excuse in that there is an acceptance that a wrong was committed, and blame is therefore justified, but the circumstances call for less blame. This concerns sentence, rather than guilt, but the integrity principle is applicable here too. In arguing that remorse should have no part to play in the trials of activists, this also extends to their sentencing. The integrity principle, with its focus upon the moral convictions and consistency of protesters, replaces remorse as a mitigating factor. In cases where the court deems a conviction appropriate, the protester’s denial that they have breached community norms but have instead acted on behalf of the community should be understood as a mitigating factor.

## 3. Cases and Methods

We explore the application of the integrity principle through the three recent, high-profile trials in English Crown Courts mentioned above. Each case was subsequently referred to the Court of Appeal (in the first two cases, the defence were the appellants; in the third case, the Attorney General sought clarification from the Court of Appeal about the scope of one strand of the defence argument).

The first case is the conviction at Preston Crown Court in August 2018 of the Frack Free Three for ‘truck surfing’, or climbing onto the cabs of lorries attempting to deliver equipment to Cuadrilla’s hydraulic fracturing site at Preston New Road, near Blackpool, blocking one carriageway of the A583 for between two and a half and three and a half days the previous summer.^69^ Charged (unusually for protest cases at that time) with the common law offence of public nuisance, two of the three were sentenced in September 2018 by HHJ Robert Altham to 16 months’ imprisonment, and one to 15 months, making this—in the words of their QC Kirsty Brimelow—the ‘first time since 1932 that environmental protesters have been given an immediate prison sentence’ in the UK.^70^ At trial, HHJ Altham had not allowed the defendants to present a plea of either duress or necessity. In their Court of Appeal interventions, both Liberty and Friends of the Earth (FoE) submitted that the sentences contravened the freedoms of belief, expression and assembly protected by the European Convention of Human Rights (ECHR) and the Human Rights Act 1998, alongside the ‘established, long-standing practice and convention’ in *Jones*^71^ that protesters ‘should be subjected to *lesser*, more lenient sentences’ because ‘their actions are motivated by genuinely-held, good-faith beliefs’.^72^ Further, for FoE, HHJ Altham’s emphasis on the activists’ ‘unswerving beliefs that they were right’ in his justification of custodial sentences amounted to an ‘impermissible criminalisation of belief’.^73^ The defendants appealed against their sentences, which were overturned in the Court of Appeal as ‘manifestly excessive’; the Court imposed instead two-year conditional discharges in recognition of time already served.^74^ Burnett LCJ drew upon *Jones*,^75^ noting that ‘well intentioned protesters are given considerable leeway … this is a consistent theme that has been running for a very long time’.^76^

The second case is the conviction at Chelmsford Crown Court in February 2019 of the Stansted 15. In March 2017, the activists cut through Stansted airport’s perimeter fence and locked themselves around the nosewheel and behind the port wing of a parked Titan Airways Boeing 757, chartered by the Home Office to deport 60 people to west Africa. The action successfully stopped the boarding and departure of the flight. Initial charges of aggravated trespass were escalated by the CPS, with the consent of the Attorney General, to a terrorism-related charge of endangering the safe operation of an aerodrome.^77^ For the Stansted 15’s solicitor, Raj Chada, ‘Never in the twenty years that I’ve practised, without any new evidence coming to light, has someone reviewed a case and moved from a charge with a maximum penalty of three months to one of life’;^78^ for the political commentator Yanis Varoufakis, this heralded a ‘brutal turn in Western democracies’.^79^ An initial trial in March 2018 ended after one week when HHJ Christopher Morgan discharged the jury; a second trial in autumn 2018 lasted 10 weeks. Here, HHJ Morgan delayed ruling on defences until after the closing of the defence case, thereby allowing the defendants to adduce evidence in support of *duress*, before ruling that it could not be put to the jury. Following the jury’s guilty verdict, Morgan imposed relatively light sentences, explicitly recognising the sincerity of the defendants.^80^ As noted above, the convictions were overturned on appeal,^81^ but the legal directions on the inapplicability of necessity were upheld, ruling that Hoffmann’s observations in *Jones*^82^ were not *obiter*, as previously thought, but the *ratio* of the judgment.

The third case is the acquittal of the Colston 4, after a 10-day trial from December 2021 to January 2022. All four were charged with criminal damage for toppling the statue of slave trader Edward Colston during a Black Lives Matter protest in Bristol on 7 June 2020.^83^ Three of the defendants were accused of pulling down the statue; the fourth of rolling the statue to the river Avon and dumping it in the harbour. Charged with criminal damage, the defence argued variously that: their act did not cause damage (as the cultural and monetary value of the statue increased due to their action);^84^ they had a lawful excuse for their actions (they believed they had the consent of the owner);^85^ they acted to prevent a crime (the statue was a public display of indecent matter^86^ and a display of a visible representation which is abusive,^87^ and Bristol City Council’s (BCC) inactions to remove or contextualise the statue constituted the offence of misconduct in public office^88^); and, following *Ziegler*,^89^ the defence of lawful excuse should be interpreted so as to assess whether a conviction would be a disproportionate interference with the defendants’ ECHR article 9 and 10 rights to freedom of conscience and expression. While this defence was put to the jury, the Court of Appeal, in response to an Attorney General’s reference, ruled it should not have been.^90^ The trial judge, HHJ Peter Blair, allowed the defence to call a series of expert witnesses to substantiate their claims. On 5 January 2022, after deliberating for just under three hours, the jury delivered a majority verdict acquitting all four defendants.

Our analysis combines several methodological approaches. For discussion of the Stansted 15 and Colston 4 trials, our approach is ethnographic, primarily based on trial observations. As noted above, in the Frack Free Three trial, the trial judge ruled out the necessity and duress defences; here, our analysis focuses primarily on the Court of Appeal’s ruling.^91^ Alongside observation, we deploy a range of related ethnographic methods, including formal semi-structured interviews with seven of the Stansted defendants;^92^ informal^93^ conversations with supporters, defendants and legal representatives during the Stansted trial; and off-the-record interviews with two members of the Stansted legal team and three members of the Frack Free Three legal team. We also participated in or observed a series of events and workshops organised by or involving a number of the Stansted defendants and legal representatives from both trials.^94^ We secured ethical clearance from Aston University, following the British Sociological Association’s guidelines;^95^ following our ethical protocol, we fully attribute statements produced in court and in open public meetings; anonymise statements gathered from interviews; and do not use off-the-record statements. In what follows, we first discuss the Court of Appeal’s ruling in the Frack Free Three case, before turning to the Stansted 15 and Colston 4 trials.

## 4. Remorse and the Frack Free Three

In the trial of the Frack Free Three, remorse was central to HHJ Altham’s imposition of custodial sentences and the Court of Appeal overturning these sentences. Burnett LCJ noted that the trial judge drew on three reasons: the extensive harm caused by the protest; the continuation of the protest despite the evident severity of the disruption; and the defendants’ lack of repentance and ongoing justification for their protest.^96^ HHJ Altham rejected any suggestion that a meaningful apology was offered, as it was ‘a submission which had been made on their behalf’.^97^ HHJ Altham found instead that each defendant:

remains motivated by an unswerving confidence that they are right and it was plain that during the course of their evidence at trial that they felt even then that they were justified in how they acted. Whilst they each make protestations of remorse those came only after they were convicted.^98^

For HHJ Altham, this ‘belief in their own correctness’^99^ constituted an attempt to justify transgressive action, so *Jones*^100^ did not apply. As noted above, both Liberty and FoE submitted that treating the appellants’ failure to renounce their views as an aggravating factor ‘has an obvious chilling effect. It is unlawful.’^101^ Counsel also claimed that it was paradoxical to invoke these beliefs when sentencing while not allowing the appellants to present these beliefs at trial.^102^

The Court of Appeal, drawing on Lord Hoffmann’s reasoning in *Jones*,^103^ rejected the argument that, as a matter of law, non-violent protesters should never be imprisoned, arguing instead that there are ‘no bright lines’, and the seriousness of public nuisance meant that the appellants had not kept their side of what we term ‘Hoffmann’s bargain’, which also imposed a requirement that protesters act ‘with restraint’.^104^ However, the Court rejected HHJ Altham’s conclusion that the defendants were likely to reoffend; the Court drew on the appellants’ pre-sentencing reports, where two of them accepted their action was ‘unreasonable and irresponsible’, and emphasised their deep regret. One defendant, Loizou, stated that he ‘disavowed’ the view that he was supporting the local community, having ‘listened to exactly how various people had been impacted’.^105^ As a result, ‘he expressed remorse for those he harmed’.^106^ Similarly, a second defendant, Roberts,

stated that after hearing the evidence from during the trial he felt guilt and remorse for their inconvenience and admitted he was naïve, not understanding the consequences of his actions at the time but has had time to reflect … He asserts that prior to the verdict, he had already made a decision to move away from working with the protest group.^107^

Liberty and FoE submitted that the sentences were excessive given the nature of the public nuisance caused and that they breached ECHR rights. The Court of Appeal ruled the sentences ‘manifestly excessive’, in a judgment loudly welcomed as a victory for protest rights. Yet, the Court of Appeal did not hold the sentences to be inappropriate *per se*, maintaining the offence to be serious; nor that HHJ Altham was axiomatically wrong to treat the activists’ persistence in their political beliefs as an aggravating factor. Rather, HHJ Altham had not recognised the defendants’ personal transformation through the trial process, their remorse for what they had done and their disavowal of their committed (and potential future) direct action. For the Court of Appeal, the defendants had accomplished a remorseful separation of act and being, and it was *this* that justified their entitlement to mitigation. As we noted above, the integrity principle respects protester’s claims that they deny wrongdoing but instead act on behalf of the community as citizens. Remorse is not, therefore, appropriate, with the integrity principle standing in for remorse. The focus here, therefore, upon remorse, is an incorrect turn in the Court of Appeal’s reasoning; the sentences should have been overturned not on the basis of remorse, but in recognition of the excessive character, given the integrity demonstrated by the defendants.

## 5. Duress, Necessity, and the Stansted 15: Policing the Political

The Stansted trial offers a different type of transformative dynamic. Here, the defendants did not express remorse or contrition, but instead sought to argue their actions were morally legitimate. In statements given under caution in police interviews after arrest, the defendants cited their belief in the serious threat to people on the flight, and their own moral duty to act. The following is typical:

I believe the deportations that were being conducted were not carried out in a lawful manner. I acted in the way I did because I genuinely and reasonably believed that the individuals on the plane were at risk of death or serious injury if returned. Bearing in mind the risk to those individuals, I acted in a reasonable and proportionate way throughout.^108^

Variations of this were also provided in court: ‘It was clear that people were in genuine peril, and I felt compelled and it was necessary to do something about it’ (Smoke); ‘I believe I was doing what I had to do in order to protect people who I was gravely concerned about’ (Clayson).^109^

In their testimonies, the defendants maintained their action did not violate collective norms, and was both morally and legally permissible. At trial, arguments over duress concerned the imminence of risk and the limits of the political, with HHJ Morgan actively policing the boundary between political and non-political statements, intervening to circumscribe the ways in which the defendants sought to justify their action. Here, the prosecution argued that rather than acting out of concern for those due to be deported, the defendants’ primary motivation was political, and to attract media publicity for their cause.

The prosecution’s cross-examination of Clayson illustrates this strategy. While the prosecutor stated that ‘this action is a political protest, isn’t it?’, Clayson responded by stating that ‘it was a direct action to stop that flight from taking off’. The prosecutor also highlighted the T-shirts worn by the defendants (which were ‘brandishing your own personal political opinions’) and the *political* protest songs they sang. Clayson responded by stating she agreed with the sentiments, ‘but that’s not why I was there’. Similarly, when asked if it was ‘necessary to break the law’, Clayson put this necessity in terms of avoiding risk: ‘if you believe someone’s life to be at risk, that should take preceden[ce] [over the law]’.^110^

What we see here is consistent with the approach adopted throughout by defence and prosecution. In Clayson’s cross-examination, the prosecution claimed this was a *political* act; Clayson, mindful of the constraints placed by HHJ Morgan on the precise terms in which the defendants could give an account of their action, disassociated the action from the group’s wider political critique of bordering and Home Office policy, stressing instead imminent harm.^111^ Similarly, in cross-examination of Evans, the prosecution questioned the defendant’s understanding of the rule of law, and charged the group with taking the law into their own hands; Evans disassociated the group from a wider critique of democratic arrangements: ‘This was never about politics, it was about people, about safety.’^112^

The defendants occasionally pushed the boundaries of this dissociation. Evans, who self-represented, was warned by HHJ Morgan that he would give ‘a degree of latitude’, but that she must only present evidence related to her personal knowledge and understanding: ‘If it appears to me that what is being said is a political statement, I will intervene … There is a line—if I think you are approaching that line, I will inform you.’^113^ Evans then built a discussion of well-known deportation cases—Isa Muazu, Adaronke Apata, Jimmy Mubenga—from her personal experience and contacts: knowing about Muazu through her friend and flatmate, a caseworker for Detention Action; becoming aware that people were deported whilst awaiting appeal, through an immigration lawyer friend; with friends, going to Apata’s High Court appeal hearing; reading a Corporate Watch report; participating in a protest at a detention centre and at Stansted in January 2017; deciding to act after hearing from Detained Voices that the Home Office had sent out deportation letters; and receiving the powerful testimony of a lesbian woman, detained in Yarl’s Wood and due to be deported on the forthcoming flight, of her fears that her ex-husband would kill her when she arrived in Nigeria.^114^ Evans read the short testimony, before telling the court: ‘I feared that when she stepped off that plane she might be killed … I felt that I had to act to prevent harm from coming to these people.’^115^

This personalised narrative is not completely shorn of its wider context. Many of the defendants spoke fluently, particularly during examination in chief, of their activism and their understanding and criticisms of the detention and deportation system. Defendants occasionally introduced wider political questions into their personal narratives, engaging in acts of *smuggling*, pushing at the boundaries policed by HHJ Morgan (for example, where defence counsel asks Evans a question about the use of chartered flights for deportations, or asks Evans to expand on her personal knowledge—‘are there any other cases where …?’, the trial judge intervenes and disallows;^116^ similarly, HHJ Morgan intervenes in defence counsel’s questioning of another defendant, Potts, ‘I’m going to stop you giving a political speech about your political beliefs’).^117^

Acknowledging this, another of the defendants told us after the trial that the emphasis on personalised physical threat should be seen in political terms, as a radical grounding of their action:

There is something … certainly, I think it’s quite, its potency comes from interweaving kind of universal truths about the sacredness of life and kind of like fundamental truth for me, that we need to fight for human dignity and respect and safety, but it’s kind of intensely political because it ties into kind of, a political critique of borders and nation states and fascism and neoliberal global capitalism so, I think it’s very important spiritually and politically to ground it in human story and human dignity and safety.^118^

Yet, as another defendant acknowledged, not talking politics in the courtroom was a conscious strategic choice taken in cognisance of courtroom power dynamics and outcomes. They acknowledged discussing the strategy, and the risk that openly discussing their political views could lead to the prosecutor responding with:

[S]o you would have done this to any plane, it wasn’t specifically about this it was just you were anti-capitalist, anti-imperialist whatever, and you see this as a racist weapon of the imperialist state and so you could have stopped any plane and it wouldn’t have mattered and all the stuff you’re talking about with the Detained Voices blog is just rubbish.^119^

The defendants, therefore, sought to narrate their action in a highly situated way, emphasising an immediate concern to prevent harm and foregrounding an ethics of care and solidarity; whilst its concern with dignity has the capacity to ‘tie into’ a much wider and more radical political critique, as the interviewee above puts it, it remains a ‘human story’ foremost concerned with safety from harm. In so doing, the defence explicitly adopted a narrow legal framing of the action, rather than a wider critique of systemic injustice; indeed, the defence explicitly stated that it was not challenging the lawfulness of the deportation process, but rather only the prevention of imminent serious harm.^120^ For the prosecution, however, these dissociated beliefs were central to the defendants’ actions. As the prosecution barrister put it:

The case cannot divorce the defendants’ actions from their political beliefs … The defendants cannot plead that their actions were out of necessity or that they were acting out of compulsion—there was no immediacy between their finding out about the situation and deciding to take action … whichever way the Court decides, the defence is fatally flawed because in a case of necessity, the defendants need to have a closer relationship with X. In reality, the defendants chose to operate the functions of the state—there is no evidence to say that they reasonably thought themselves responsible for the safety of deportees on board; they acted on behalf of their general concern … When analysed fully, the case for necessity is not available to the defendants.^121^

HHJ Morgan ruled the defence had not met the test for duress to be put to the jury, thus removing a key plank of the defence’s argument.^122^ It is important to underline that throughout, the defences of necessity and duress were conflated in court; even though the defendants successfully negotiated the line constantly policed by Morgan separating necessity (politics, an account, not tolerated) from duress (imminent harm, an excuse, tolerated), Morgan refused the jury the possibility of deciding on duress. While the Stansted 15 were subsequently acquitted by the Court of Appeal, Morgan’s directions on duress were upheld by the Court, which effectively endorsed this conflation of separate defences, making neither available to protest defendants.^123^

## 6. Colston 4: An Effective Communicative Endeavour?

The trial and acquittal of the Colston 4 provides a stark contrast to the previous two cases. In this trial (as in the others), the facts were, broadly speaking, not in doubt: three of the defendants had contributed to bringing down Colston’s statue; the fourth had participated in rolling the statue into the harbour. All four defendants were visible on CCTV and in other images, repeatedly played in court. Broadly, the defendants admitted their involvement, as alleged. However, the three defendants charged with bringing down the statue contested the prosecution’s case that their action was pre-planned. This ostensibly tangential detail reveals a series of important distinctions concerning the type of account that the defendants were able to give in court, as we shall discuss further below. First, however, it is important to underline that the charge of criminal damage under the CDA 1971 enables defendants to elect to be tried in a Crown Court, where the alleged damage is unknown or exceeds £5000, and to claim they had lawful excuse, and (unlike, therefore, in the previous two cases) for this defence to be put to a jury.

This is, of course, policed by the rulings of the trial judge. Here, HHJ Blair removed some defences from the jury (at the closing of the defence case), including the argument that the continued public display of the statue amounted to misconduct in public office. Those left available, however, allowed the defendants to develop a radical reinterpretation of their action. Over 10 days, they were able to switch the focus as to who was on trial and present their behaviour as a moral and civic action *on behalf of* the community.

### A. Putting Slavery on Trial

The prosecution sought to frame the trial as a simple case of criminal damage. They sought to characterise it as a ‘violent act’ and an ‘affront to democracy’; the fate of the statue, which had been the object of mounting local criticism for over two decades, was a matter for BCC, and not for the defendants to take the law into their own hands. The prosecution accepted that Colson was ‘a divisive figure’ and that it is ‘common ground that he made his fortune through the slave trade’; nevertheless, they claimed the trial was ‘not about him or his vilification, which is wholly irrelevant to the matter on which the case rests’.^124^ This claim was reiterated at the closing of the prosecution case: ‘This is a criminal trial about the actions of these four defendants; it is not a public enquiry. This trial is not about politics; it is not about emotions, it is about cold hard facts.’^125^

For the defence, in contrast, what the trial was not about was their involvement in damaging the statue; it was, rather, in the social meaning and democratic legitimacy of this act. Crucially, the availability to them of a range of defences made it possible to place their motivations centre stage. In particular, the prevention of crime defence (that the statue was indecent and abusive), the lawful excuse defence (they believed they had the consent of the owners—the people of Bristol) and the requirement for a conviction to be a proportionate interference with their article 9 and 10 rights called for an analysis of the actions of Colston, BCC and the Society of Merchant Venturers (SMV),^126^ and an explanation of their motivations, reasons and beliefs. As a consequence, the defence were able to turn the tables and place Colston, BCC and the SMV on trial.

Throughout the trial, the defence critiqued Colston’s role in the slave trade and questioned whether the statue was appropriate. One defendant, Ponsford, when giving evidence, was asked what they knew of Colston, to which he said ‘he was responsible for the enslavement and transportation of over 80,000 slaves, some of which were children. 12,000 people died on those ships.’^127^ The display of the statue was, therefore, ‘very disrespectful and offensive to the people of Bristol’.^128^ Co-defendant Willoughby said that Colston was ‘a racist and slave trader who has murdered thousands’ and the statue was ‘a monument to racism’.^129^ Another defendant, Graham, said ‘[to] have such a mass-murdering human being venerated on a pedestal literally in the heart of the city I think is disgusting’.^130^

This argument was developed not just by the defendants, but by a series of witnesses that the defence was able to call. Most prominently, this came from the expert evidence of the historian David Olusoga. In what was a *tour de force* public lecture on the history of slavery in Bristol, Olusoga placed Colston at the centre of a grim and distressing trade. Olusoga described the harms of the slave trade, and Colston’s rise in the Royal African Company. Colston’s philanthropic work was mere ‘reputation laundering’.^131^ The ‘cult of Colston’^132^ was subject to increasing questioning from the 1920s to the present day; there were numerous attempts to recontextualise the statue (through replacing the plaque) and calls to have it removed. Olusoga noted that the most recent efforts to recontextualise the statue failed due to the SMV’s attempts to ‘tone down the wording of a new plaque’.^133^ Olusoga described the SMV as ‘effectively a guild given exclusive rights over Bristol’s waterways’; while they predated the slave trade, ‘many members were slave traders and owners’ and they acted in ‘defence of the slave trade’.^134^

As a consequence, the defence were able to also place the SMV on ‘trial’, leading to the jury asking for guidance on the SMV’s role. Graham described them as ‘an undemocratic, unelected body of wealthy people who have a lot of power and influence’ and linked their abuse of power in blocking the statues’ recontextualisation with the abuse of power that led to George Floyd’s death and the Black Lives Matter movement’s rise.^135^

BCC was also criticised for its inactions concerning the statue. This was relevant to two claimed defences: the belief the owners consented to the removal of the statue; and that the defendants were preventing the crime of misconduct in public office (BCC had ignored repeated public campaigns to remove the statue or change the wording of the plaque). Ponsford, commenting on efforts to change the plaque, said ‘it was a disgrace the statue was still there’;^136^ Skuse, the fourth defendant, argued that if ‘there was racist graffiti, they’d [BCC] remove it, and they haven’t removed this statue’.^137^ Willoughby compared the statue to ‘a hate crime’.^138^ Graham accused BCC of ignoring the people: ‘I believe democracy had well and truly broken down around that statue.’^139^ In his closing address, defence lawyer Raj Chada drew the parallel directly: ‘It may be that the Council should have been on trial for this offence.’^140^

### B. Moral and Civic Action on Behalf of the Community

We have seen, above, how the defence were able to effectively place Colston, the SMV and BCC on trial. In contrast, they framed their behaviour as a moral and civic action on behalf of the whole community. Their action was symbolic; they were standing in allyship against an injustice perpetrated by the powerful and perpetuated by a failure of democracy. The consequences of their action, they claimed, were positive and benefited the whole community of Bristol.

One important way in which the defence characterised their actions as moral was through the use of symbolic imagery. Graham, commenting on the use of ropes around the statue’s head, said ‘the noosing of a slave trader would be symbolic’.^141^ Skuse, describing dumping the statue into the harbour, noted the action’s symbolic meaning. The path taken (rolling the statue over the cobbles) and the destination (Pero’s Bridge) were significant: ‘I’ve always associated cobbles as a place where people have been dragged without their consent’,^142^ while Pero was ‘an enslaved person who got his name freed after he died’.^143^ The act, therefore, was a symbolic act of moral retribution: ‘It was like a sentencing, like sentencing him [Colston] to death.’^144^

This moral character of the action was a clear feature of the advocates’ summing up: the defendants ‘did not destroy history, they created history’;^145^ ‘correcting the record is not vandalism; it is progress’;^146^ they were ‘building a better future for everyone’;^147^ ‘removing the statue has a positive impact, a healing effect’;^148^ this was ‘not an arbitrary act of vandalism’, but rather ‘a deliberate act of solidarity with all those who are oppressed by the representation of Colston’.^149^ Indeed, faced with the prosecution’s charge that the action was violent, Willoughby said it was an ‘act of love for my fellow man. An act of love, not an act of violence’ and ‘this was an act of love and solidarity’.^150^

As a consequence of seeing their act as one on behalf of the community, the defendants showed no sign of remorse for their actions. Indeed, they were unapologetic for their acts, the testimony of Skuse being a strong example of this: removing Colston ‘just felt right’ and that ‘these people [who toppled the statue] had done a great thing’,^151^ adding that they paused just before the final act of pushing it into the water because ‘it needed to be marked, say a few words of victory, and then he [Colston] can fuck off’.^152^

### C. Policing the Political, Redux

This overview leaves a nagging question for our argument. Earlier, we argued that it is important to distinguish between accounts and apologies; that accounts are explanations rooted in the moral consistency of act and being, whereas apologies recognise the exceptional nature of the act and seek to be excused on these grounds for the divergence between act and being. Necessity defences are accounts; duress defences share elements of apologies; integrity, not remorse, should be privileged in protest trials. What we have set out above is self-evidently a consistent argument put forward by defendants acting with integrity. Yet, there are strong elements of excuse in their defence. Does this matter? Perhaps not. The defendants were able to fully contextualise their action; it was, equally clearly, an exceptional action, taken after many other attempts to remove the statue had failed or been blocked, and which was directed at this statue and this statue only. In Duff’s terms, the defendants presented themselves as citizens representing their community, to their community (in the form of a jury); this was axiomatically an effective communicative event, and would equally have been so had the jury found the defendants guilty.

Yet, it is also the case that whilst the Colston 4 associated their action with community, they did not associate themselves with it as an expressly political act. Two elements of the defence should retain our attention.

First, as briefly noted above, though the defence generally accepted the facts of the alleged offence, they did not accept the prosecution’s version of those events. Central here is a complex argument about the illegitimate nature of the action. For the prosecution, the action was separate from the Black Lives Matter demonstration taking place in Bristol that day. The BLM demonstration was ‘friendly, engaging, and very much a community event’, legitimate, collective and peaceful; in contrast, the pulling down of the statue was the violent act of a small minority, ‘a criminal activity separate from [the march]’.^153^ Moreover, the action was *planned*; in cross-examination of Ponsford and Graham, the prosecution repeatedly queried the length of the 30-metre ropes brought by each. In response to Graham’s claim that ‘It’s honestly the only piece of rope that I have’, the prosecutor responded that a ‘longer rope of course would allow more people to grab hold of it, would allow more individuals to participate in pulling the statue down’, later noting that ‘a shorter rope would have done the same job for the noose, symbolically; but this would not have allowed so many people to pull on the rope’.^154^

Both Graham and Ponsford denied the implied intentionality: there was no real planning beforehand, just a brief conversation between them the night before; it was only when they found themselves at the statue that each realised the other had brought a long rope (‘I can categorically tell you that never did we set a time to meet, that we did not agree to bring a rope … I had no idea that [Ponsford] was going to bring a rope’);^155^ they did not expect the statue to fall; the act was only intended to be symbolic; the defendants could not have done it alone, nor would they have wanted to; Graham and Ponsford brought ropes so the people of Bristol could pull on them if they wanted to. This was, in other words, a kind of ‘collective effervescence’ (in Durkheimian terms), where the community came together in spontaneous action. It was not a criminal act, but a legitimate expression of collective community feeling. But neither was it accepted by the defence to be a *deliberate*, organised act.

Second, the defence repeatedly stressed the defendants’ grounding in the community. Graham, for example, described the act as one of allyship on behalf of the community. When denouncing racism, Graham suggested that ‘I had been a terrible ally’, while ‘privilege’ created a duty to ‘stand in solidarity for black lives’,^156^ and removing the statue was important as it was ‘one of those things that seeks to delegitimise the Britishness of black people’.^157^ Importantly, whilst closing by underlining the importance of protest, defence counsel emphasised that the defendants were not ‘activists’; the ‘four on trial are not professional protesters or troublemakers, not vandals bent on mindless destruction’.^158^ Graham was explicit on this point: ‘to do something like that was very out of character for me. Like I said I was not an activist, pulling statues down in the middle of the city centre is not who I am.’^159^

This is an odd thing for someone to say who went prepared with a rope to pull down a statue, pulled down the statue and consistently argued in court that it was morally right to have pulled down that statue. It is, in distilled form, the narrative structure of an apology, an attempt to distance oneself from the infringement, to separate act and being. In both the words of counsel and the defendant, it is a separation of the legitimate actor, acting exceptionally, from the illegitimate actor, the professional protester, the activist, the troublemaker, acting with prior planning and with the will and capacity to act in this way *more than once*.

## 7. The Integrity Principle and the Protest Trial as a Communicative Endeavour

These three trials shed light on the extent to which the protest trial is, in Duff’s terms, an effective communicative endeavour. We noted, following Duff, how the trial is more than an adjudicative process, with non-epistemic values being important; and that for trials to fulfil their discursive function, defendants must be able to give an account of their action in ways that make sense to them and the law, through the provision of appropriate defences. We also argued that protest trials should, normatively speaking, be governed by an integrity principle, privileging the moral consistency of the defendants in a way that does not enforce a separation between action and being, and enabling them to present as discursive actors with sincerity. The three factors we identified as central to the principle are: defendants should not have to show remorse, but instead be able to attempt to justify their acts in *law* (otherwise all that is left is conscience, with no means of expressing this adequately); that they be able to claim that they acted on behalf of the community; and that they be able to articulate their philosophical beliefs.

The picture that emerges from the three cases we discuss here is, however, messy. In the Frack Three Free case, we see a clear denial of the trial as a communicative enterprise: at trial, HHJ Altham ruled the presentation of any form of account from the defendants to be inadmissible; and in sentencing the defendants to prison included mention that they had failed to adequately repudiate their own actions. In the Stansted 15 case, the defendants were able to account for their actions, though within tightly defined limits. Here, normative distinctions between necessity and duress were blurred in practice, as counsel (for both defence and prosecution) and judge used the terms interchangeably. Yet admissibility rulings throughout the trial reduced the discursive space available to the defendants. In their defence, the Stansted 15 gave a moral justification, or *account*, of their action, not an *apology* or excuse for it. Yet, this account *nonetheless* proceeded by ‘transforming what could be seen as offensive into what can be seen as acceptable’.^160^

Under the shadow of very severe potential sentences and HHJ Morgan’s active boundary policing, the defence strategy minimised the overtly political in favour of a personalised ethics of care and concern. This reliance on a defence foregrounding imminence of harm structurally divested the *political agency* of the defendants: in the terms of their defence, they acted not because of their collective and politically transformative commitment, but because of the duress of immediate circumstances, privileging a normative human story over a potentially transgressive and system-challenging discourse. Further, attempts by defendants to smuggle political critique into their accounts provided the rationale for HHJ Morgan to prevent duress (‘necessity’, as he termed it) being put to the jury. The trial process thus forced the defendants to perform a separation of act and being, to dissociate their political selves from their action. This separation, however, did not lead to acquittal, an option HHJ Morgan effectively removed from the jury; rather, it was a route to conviction, associated with leniency in sentencing.

Neither trial, therefore, provided an opportunity to ‘put the law, and the polity whose law it is, on display, and thus [open] the way to critical reflection’.^161^ These trials did not treat the defendant as a member of a moral community with shared values, and the defendants were not fully able to express, in their voice, their values and reasons for acting. The law was not, therefore, a ‘“common” law’ that reflects the ‘values that we share as members of a political community’.^162^

There are nonetheless differences in these cases. The Stansted 15 offered no remorse for their action, yet this did not impact on sentencing, as HHJ Morgan followed the convention of leniency for direct action protesters. In contrast, HHJ Altham’s decision to impose custodial sentences on the Frack Free Three, and focusing, when doing so, upon a lack of remorse, upheld the general rule for ordinary trials, but directly contravened both the integrity principle and the convention for direct action protesters as set out in *Jones*.^163^ This was apparently corrected by the Court of Appeal, but *not in the terms set out by Hoffmann*; rather than finding that HHJ Altham was wrong to consider remorse (and should instead have had due regard to the sincerity of the defendants’ commitment), the Court applied a lesser sentence on the basis that HHJ Altham had been wrong to doubt the sincerity of their remorse. The Court’s ruling therefore suggests that HHJ Altham was in fact right in principle: only by repudiating their political beliefs were the three convicted activists suitable for release from prison. Rather than protecting acts of political expression, the outcome requires their abnegation, an apology for them.

Our third case provides something of a contrast. The Colston 4 trial provided a very different space for the defence to account for their actions and motivations. They rebutted the prosecution’s characterisation of the toppling of the statue as a violent and anti-democratic act; instead, they maintained it was an act of solidarity, a moral and civic action by and on behalf of the community. Colston, the SMV and BCC were all effectively placed on trial, turning the tables. The capacity of the defendants to do this was enabled through a combination of numerous elements: the availability of a lawful excuse defence; the relatively light potential sentences faced by the defendants if found guilty, which reduced their risk; the existence in the city of strong political and activist networks, which demonstrated community support; and the trial management of HHJ Blair, who allowed the defence to call multiple witnesses. Here, we argue, the protest trial worked as it should: as an effective communicative endeavour privileging the integrity principle.

Indeed, there was no display of remorse in the accounts advanced by the Colston 4 (Skuse, as noted above, described their actions as a ‘victory’ and ‘a great thing’^164^); instead, they focused upon the positive benefits for the community in removing a name that was a stain on the streets and institutions of Bristol. In characterising their actions in this way, there was only a limited separation between act and being; the only hints at this separation concern the avowed spontaneity of their actions (in stark contrast, for example, with the Stansted 15, where Evans, as noted above, openly set out the timeline of events), and their self-presentation as members of the community, rather than as activists. Indeed, whilst the defendants were able to fully account for their motives and values in acting as they did, they rejected the claim that they were ‘activists’. Part of this distancing may well have been a tactical claim, though we have no reason to doubt the sincerity of the accounts given by the defendants; in court, they said they had no history of direct action protest (and only limited history of participating in demonstrations). Nonetheless, the effect of this account is to frame activism, as a sustained commitment that requires planning, organisation and experience, as outside of the community, rather than integral to societal change.

It is worth outlining, therefore, what kind of political subject is worthy of being heard in these cases. We are, clearly, dealing with a limited number of cases which have exceptional features; but leniency at sentencing in the first two cases suggests that *freedom of political belief* remains recognised in Hoffmann’s bargain in *Jones*^165^ as a mitigating factor. Nonetheless, all three cases suggest that the *translation of belief into political action* is considered an aggravating factor in judicial decision making, given the importance of disavowal for the release from prison of the Frack Free Three, the policing of political accounts in the Stansted 15 trial and the separation between ‘activism’ and ‘moral community action’ in the Colston 4 trial. Although it superficially appears a victory for protest rights, the Frack Free Three appeal ruling is especially worrying: not only does it not protect activists with prior histories of action, but it also requires activists to show remorse in order for leniency to be afforded. Further, we suggest that the effect of ‘manifestly excessive’ charging (in the first two cases) and initial (for the Frack Free Three) and potential (Stansted 15) sentencing effectively places defendants in a position where they have few options but to divest their accounts of their political imaginary, such is their risk.

As we have seen, the defendants are constrained to be sincere, but non-political, with experience of high-risk activism and consistent political beliefs considered as disqualifying factors. They must, above all, be non-transgressive, be motivated by caring and avoid systemic critique. This is, plainly, a figure riven with contradictions. Moreover, as the Stansted 15 case shows, where activists have the experience and understanding of risk that enables action to be taken where potential harm is imminent, this is where we are most likely to see excessive charging and the imposition of severe process and/or outcome penalties. Even the intervention by FoE at the Frack Free Three appeal, on behalf of the defendants, explicitly emphasised that the three defendants had no prior convictions, and represented a low risk of reoffending. Here, therefore, the logical conclusion of the argument is that activists with prior direct action protest convictions would not benefit from leniency at sentencing. In other words, sincerity of belief is protected for activists breaking the law—as long as they only do it once.

## 8. Conclusions

The argument developed in these pages makes five important contributions to our understanding of the relationship between criminal justice, direct action protest and the protection of philosophical belief in the English and Welsh courts.

First, we follow Duff, arguing the trial should go further than mere adjudication and should be a communicative enterprise where the defendant is held to account by the community. We make the first attempt to apply this reasoning to the trials of direct action protesters, which we consider a separate category of trials. Normatively, we argue that protest trials are distinct from ordinary trials, rejecting the applicability of the remorse principle to these trials. Instead, protest trials should operate on the integrity principle, privileging moral consistency and enabling the defendant to account for their actions in ways that make sense to them, without divesting their political selves, and to do so in terms of a legal defence. The trial should, therefore, allow defendants to account in ways that are morally consistent with their philosophical beliefs and, in cases where the court deems a conviction appropriate, the absence of remorse should not mean a loss of mitigation, but instead the moral integrity of the activist acts as a mitigating factor. It is important that the accounts these defendants are able to give in court authentically reflect their motivations and contextualise their actions, and are available in *law*. A criminal justice process which denies this possibility fails this test.

Second, we apply this normative framework to the analysis of three recent, high-profile cases, each with significant consequences for the public sphere. As we have shown, the separation of act and being that results from the particular forms of remedial work observed in these trials operates in ways that deny defendants the opportunity to account, authentically, for their action. Defendants are not, therefore, treated as full members of the community and possible shared values are supressed. In the Frack Free Three and Stansted 15 cases, the trial process fails the test. The trial of the Colston 4 provides a contrast, showing how the availability of justificatory defences allows for a truly communicative trial where the defendants are able to place their values and motivations before the court and address the jury as members of the community, acting on behalf of the community in accordance with shared values. The Colston 4 trial also allowed defendants to question the standing of the community to hold them to account, pointing to failures of democracy and the political process which led to injustice. This trial, therefore, offers a glimpse of a conceptually coherent way of conducting protest trials. Nevertheless, there remains a form of separation even here, with ‘activism’ consciously separated from action.

Third, we argue that, despite their apparently lenient outcomes, the conditions of charging, the terms of the Court of Appeal’s judgment with respect to remorse (in the Frack Free Three case) and the conduct of the trial with respect to necessity (in the Stansted 15 case) effectively narrow the potential expression of protest rights and challenge the protection of philosophical belief supposedly enshrined by *Jones*.^166^ As such, these prosecutions and their outcomes should be considered serious acts of the chilling of dissent. Although the Colston 4 were acquitted, and they were able to place Colston, BCC and the SMV in the dock, the Court of Appeal’s subsequent conclusions suggest this was an exceptional occurrence. In ruling that lawful excuse should have been interpreted so as not to include the defendants’ ECHR rights and hinting that the prevention of crime defence should also have not been put to the jury,^167^ the Court of Appeal effectively reduces the scope for a similar defence in the future.

Fourth, we argue that the distinction between duress and necessity, commonly elided in practice, is in fact consequential in protest trials, as it affects the types of accounts these defendants may make in court, and thus the effectiveness of the trial as a communicative endeavour. As we have seen, necessity and duress have distinct and opposing structural and symbolic properties: whilst necessity is justificatory, duress is excusatory. In the Stansted 15 trial, the defendants did not disavow their action, or seek to excuse it; yet, the availability in law only of an argument based on compulsion to act through the threat of imminent harm meant that they were unable to mount a defence based on the wider social harm of the detention and deportation system. We believe that it would have been of wide collective benefit for the defendants to have been able to present this sort of account in court, explaining their action in terms that fully made sense to them and motivated them to do it. Instead, they were obliged to undertake a form of remedial work based on a separation of act and being.

Finally, we suggest that sustained observational analysis of trials, particularly from an ethnographic and micro-sociological perspective, is revealing of the practical operation of the judicial dynamics at play in the ‘extraordinary’ trials and court rulings of social movement actors in the English and Welsh courts, and of the gap between how protest trials ought to function and how they do. In doing so, we begin to outline how the protest trial is a distinctive form of trial, which is dependent for its legitimacy on the integrity principle. Understanding questions of justification and excuse, remorse and integrity, of whether and how processes of separation of act and being and remediation are performed and imposed can be expected to be central to practical considerations of the protection of protest rights in the courts. In the context of widespread concern over the chilling of dissent in the UK, we suggest that this agenda is an urgent one.

## Footnotes

1 See eg Paul Roberts, *Roberts and Zuckerman’s Criminal Evidence* (3rd edn, OUP 2022).

2 For simplicity we will, throughout the article, refer to the protest ‘trial’, as most of our focus here will be on trials, but as we will see, the integrity principle extends to sentencing (and further work could also show how it extends to other parts of the criminal justice process).

3 Antony Duff, *Answering for Crime: Responsibility and Liability in the Criminal Law* (Hart Publishing 2007).

4 Erving Goffman, *Relations in Public: Microstudies of the Public Order* (Pelican 1971) 139.

5 Stephanos Bibas and Richard A Bierschbach, ‘Integrating Remorse and Apology into Criminal Procedure’ (2004) 114(1) Yale LJ 85; Richard Weisman, ‘Being and Doing: The Judicial Use of Remorse to Construct Character and Community’ (2009) 18(1) Social & Legal Studies 47; Joane Martel, ‘Remorse and the Production of Truth’ (2010) 12(4) Punishment & Society 414; Susan A Bandes, ‘Remorse and Criminal Justice’ (2016) 8(1) Emotion Review 14; Irene van Oorschot, Peter Mascini and Don Weenink, ‘Remorse in Context(s): A Qualitative Exploration of the Negotiation of Remorse and its Consequences’ (2017) 26(3) Social & Legal Studies 359.

6 B Douglas Robbins, ‘Resurrection from Death Sentence: Why Capital Sentences should be Commuted upon the Occasion of an Authentic Ethical Transformation’ (2001) 149(4) U Pa L Rev 1115, 1134.

7 Michael Proeve and Steven Tudor, *Remorse: Psychological and Jurisprudential Perspectives* (Ashgate 2010), 48.

8 Richard Weisman, *Showing Remorse. Law and the Social Control of Emotion* (Ashgate 2014).

9 ibid 36 and 41.

10 Above (n 5).

11 Sentencing Council, *General Guideline: Overarching Principles* (2019). <www.sentencingcouncil.org.uk/overarching-guides/magistrates-court/item/general-guideline-overarching-principles/> accessed 6 August 2023.

12 Hannah Maslen, ‘Penitence and Persistence: How Should Sentencing Factors Interact?’ in Julian V Roberts (ed), *Exploring Sentencing Practice in England and Wales* (Palgrave 2015) 183. See also Julian V Roberts, ‘Punishing Persistence: Explaining the Enduring Appeal of the Recidivist Sentencing Premium’ (2008) 48 Brit J Criminol 468.

13 Steven Keith Tudor, ‘Why Should Remorse be a Mitigating Factor in Sentencing?’ (2008) 2(3) Criminal Law and Philosophy 241, 241–2.

14 Brian Doherty and Graeme Hayes, ‘Having Your Day in Court: Judicial Opportunity and Tactical Choice in Anti-GMO Campaigns in France and the United Kingdom’ (2014) 47(1) Comparative Political Studies 3; Isabelle Sommier, Graeme Hayes and Sylvie Ollitrault, *Breaking Laws: Violence and Civil Disobedience in Protest* (Amsterdam UP 2019).

15 Kimberley Brownlee, *Conscience and Conviction: The Case for Civil Disobedience* (OUP 2012) 18.

16 Steven M Bauer and Peter J Eckerstrom, ‘The State Made Me Do It: The Applicability of the Necessity Defense to Civil Disobedience’ (1987) 39(5) Stan L Rev 1173, 1173.

17 Brownlee (n 15).

18 Christopher Bennett, ‘The Role of Remorse in Criminal Justice’ in Michael Tonry (ed), *Oxford Handbook Online in Criminology and Criminal Justice* (OUP 2016) (no pagination).

19 Brownlee (n 15) 9.

20 David Mead, *The New Law of Peaceful Protest. Rights and Regulation in the Human Rights Act Era* (Hart Publishing 2010) 25ff.

21 [2006] UKHL 16, [2007] 1 AC 136.

22 Peter Thornton and others, *The Law of Public Order and Protest* (OUP 2010) 355–8.

23 As we are developing the integrity principle and its application as a new idea, we acknowledge that some of its elements could be developed further and we plan to do so in further work.

24 As this is a mapping exercise, how such reform occurs (by the development of the common law, through legislative amendments or, for sentencing, the addition of protest specific guidelines) is outside the scope of this article.

25 See Steven Cammiss, Graeme Hayes and Brian Doherty, ‘Necessity, Non-Violent Direct Activism, and the Stansted 15: Reasserting “Hoffmann’s Bargain”’ (2002) 85(6) MLR 1515.

26 Roberts (n 1).

27 Antony Duff and others, ‘Introduction: Towards a Normative Theory of the Criminal Trial’ in Antony Duff and others (eds), *The Trial on Trial Volume One: Truth and Due Process* (Hart Publishing 2004) 22 and 23.

28 Antony Duff, *Trials and Punishment* (CUP 1986) 142.

29 Duff (n 3) 23.

30 ibid 28.

31 ibid 86.

32 ibid 49.

33 ibid 45 and 46.

34 ibid 7.

35 ibid 192.

36 Goffman (n 4) 138.

37 ibid 139.

38 ibid 144.

39 Nicholas Tavuchis, *Mea Culpa. A Sociology of Apology and Reconciliation* (Stanford UP 1991) 18.

40 John Alan Cohan, ‘Civil Disobedience and the Necessity Defense’ (2007) 6(1) Pierce Law Review 111, 111.

41 Shaun P Martin, ‘The Radical Necessity Defense’ (2005) 73(4) U Cin L Rev 1527, 1552–3.

42 ibid 1530.

43 ibid 1554–5.

44 Tavuchis (n 39).

45 James L Cavallaro Jr, ‘The Demise of the Political Necessity Defense: Indirect Civil Disobedience and United States v Schoon’ (1993) 81(1) CLR 351; William P Quigley, ‘The Necessity Defense in Civil Disobedience Cases: Bring in the Jury’ (2003) 38(1) New Eng L Rev 3, 41–9; Stephen S Schwartz, ‘Is There a Common Law Necessity Defense in Federal Criminal Law?’ (2008) 75 U Chi L Rev 1259, 1271–2; Lance N Long and Ted Hamilton, ‘The Climate Necessity Defense: Proof and Judicial Error in Climate Protest Cases’ (2018) 38 Stan Envtl LJ 57, 106–7.

46 Hugo Tremblay, ‘Eco-terrorists Facing Armageddon: The Defence of Necessity and Legal Normativity in the Context of Environmental Crisis’, (2012) 58(2) McGill LJ 321; Long and Hamilton (n 45) 62–8; Joseph Rausch, ‘The Necessity Defense and Climate Change: A Climate Change Litigant’s Guide’ (2019) 44(2) Colum J Envtl L 553.

47 Rausch (n 46) 569–70.

48 Trial of activists who had damaged military aircraft at British Aerospace’s Warton factory.

49 Trial of activists who had broken into E.On’s Kingsnorth coal-fired power station.

50 For these trials, see Doherty and Hayes (n 14); Graeme Hayes, ‘Negotiating Proximity: Expert Testimony and Collective Memory in the Trials of Environmental Activists in France and the UK’ (2013) 35(3) Law & Policy 208; Brian Doherty and Graeme Hayes, ‘Tactics, Traditions, and Opportunities: British and French Crop-Trashing Actions in Comparative Perspective’ (2012) 51(4) European Journal of Political Research 540; Sommier, Hayes and Ollitrault (n 14).

51 Above (n 21).

52 Potentially, the defence of duress of circumstances was still available; see Simon Gardner, ‘Direct Action and the Defence of Necessity’ [2005] Crim LR 371. However, in *Thacker* [2021] EWCA Crim 97, [2021] 2 WLR 1087, the Court of Appeal treated the defence of duress of circumstances as synonymous with necessity and denied that this defence was available, on the facts, for the Stansted 15. See Cammiss, Hayes and Doherty (n25).

53 Above (n 21) [89].

54 For the CDA 1971.

55 For all other offences. Duress of circumstances, developed from duress, applies where a defendant reasonably acts to avoid an imminent threat of death or serious injury.

56 eg Pamela Hobbs, ‘“Is *That* What We’re Here About?”: A Lawyer’s Use of Impression Management in a Closing Argument at Trial’ (2003) 14(3) Discourse & Society 273; Susan A Bandes, ‘Empathetic Judging and the Rule of Law’ (2009) 31(1) Cardozo L Rev De Novo 133; Michael J Higdon, ‘Oral Argument and Impression Management: Harnessing the Power of Nonverbal Persuasion for a Judicial Audience’ (2009) 57 Kansas Law Review 631; PA Zoettl, ‘Let Justice be Done: A Performative View on Portuguese Criminal Trial Procedures’ (2016) 13(4) Communication and Critical/Cultural Studies 400; Lisa Flower, ‘Doing Loyalty: Defense Lawyers’ Subtle Dramas in the Courtroom’ (2018) 47(2) Journal of Contemporary Ethnography 226.

57 Paul Rock, *The Social World of an English Crown Court. Witness and Professionals in the Crown Court Centre at Wood Green* (Clarendon Press 1993); Thomas Scheffer, *Adversarial Case-Making: An Ethnography of English Crown Court Procedure* (Brill 2010); Jessica Jacobson, Gillian Hunter and Amy Kirby, *Inside Crown Court: Personal Experiences and Questions of Legitimacy* (Policy Press 2015).

58 Alan Norrie, *Crime, Reason and History. A Critical Introduction to Criminal Law* (3rd edn, CUP 2014) 201.

59 ibid, 218.

60 Cammiss, Hayes and Doherty (n 25).

61 CPS Legal Guidance, ‘Defences—Duress and Necessity’ (19 October 2018). <https://web.archive.org/web/20240520213812/www.cps.gov.uk/legal-guidance/defences-duress-and-necessity> accessed 18 February 2025.

62 Norrie (n 58).

63 George P Fletcher, ‘Should Intolerable Prison Conditions Generate a Justification or an Excuse for Escape?’ (1979) 26(6) UCLA L Rev 1355; George P Fletcher, ‘Rights and Excuses’ (1984) 3(2) *Criminal Justice Ethics* 17; George P Fletcher, ‘The Right and the Reasonable’ (1985) 98 Harv L Rev 949.

64 Fletcher, ‘Should Intolerable Prison Conditions Generate a Justification or an Excuse for Escape?’ (n 63) 1358.

65 Fletcher, ‘Rights and Excuses’ (n 65) 19.

66 Joshua Dressler, ‘Foreword. Justifications and Excuses: Brief Review of the Concepts and the Literature’ (1987) Wayne L Rev 1155, 1163.

67 Schwartz (n 45) 1264–5; Ian Howard Dennis, ‘On Necessity as a Defence to Crime: Possibilities, Problems and the Limits of Justification and Excuse’ (2009) 3 Criminal Law and Philosophy 29, 32.

68 Norrie (n 58) 215.

69 A fourth activist who participated in the action pleaded guilty and was handed a 12-month suspended custodial sentence.

70 UCL public meeting, ‘Counter-Terror Law and Practice: A “Crushing Blow” for Protest Rights?’ (UCL Centre for Access to Justice Student Pro Bono Committee, 1 March 2019) (fieldnotes). The claim is relatively controversial, particularly for environmental protesters jailed in anti-roads protests in the 1990s, though Brimelow’s formulation differentiates this conviction from prison sentences for such reasons as non-payment of fines or breaching injunctions.

71 Above (n 21).

72
*Roberts* [2018] EWCA Crim 2739, [2019] 1 WLR 2577, [30].

73 Submission to the Court of Appeal on behalf of Friends of the Earth Limited, R v Loizou, Blevins and Roberts, 15 October 2018; Submission to the Court of Appeal on behalf of Liberty, R v Loizou, Blevins and Roberts, 15 October 2018.

74 Fieldnotes, Court of Appeal, 17 October 2018; *Roberts* (n 72) [2].

75 (n 21).

76 Fieldnotes (n 74).

77 Aviation and Maritime Security Act (AMSA) 1990, s 1(2)(b). See Graeme Hayes, Steven Cammiss and Brian Doherty, ‘Disciplinary Power and Impression Management in the Trials of the Stansted 15’ (2021) 55(3) Sociology 561.

78 UCL public meeting (n 70).

79 Varoufakis, ‘Support the Stansted 15: When Solidarity Becomes a Terrorist-Related Offence Liberty and Democracy Die’ (22 March 2018) <www.yanisvaroufakis.eu/2018/03/22/support-the-stansted-15-when-solidarity-becomes-a-terrorist-related-offence-liberty-and-democracy-die/> accessed 6 August 2023. The CPS maintained the defendants ‘were not prosecuted under terrorism legislation’; see Stansted Airport case news release, 8 February 2019 <www.cps.gov.uk/cps/news/stansted-airport-case>. We consider this claim to be patently false.

80 Fieldnotes, Chelmsford Crown court, 6 February 2019.

81
*Thacker* (n 52).

82 Above (n 21).

83 Colston was a Bristol merchant and (latterly) politician in the late 17th and early 18th centuries, with a central role in the transatlantic slave trade, notably as a senior executive in the Royal African Company. Colston was a member of a powerful civic trade group, the Society of Merchant Venturers, and left a series of charitable endowments to the city; in addition to his statue, at the time of the demonstration he was commemorated on no less than 13 of the city’s street names, whilst he was represented as the Good Samaritan on a stained glass panel in Bristol Cathedral.

84 This was not put to the jury; instead, they were given a dictionary definition of damage.

85 This was put to the jury.

86 Indecent Displays (Control) Act 1981, s 1. This was put to the jury.

87 Public Order Act 1986, s 5. This was put to the jury.

88 This was not put to the jury.

89 [2021] UKSC 23, [2021] 3 WLR 179. The Supreme Court held that in assessing whether the defendant had a lawful excuse for obstructing the highway, their ECHR rights (particularly arts 10 and 11) should be included in a proportionality exercise to assess whether a conviction would be an adverse interference with those rights.

90
*Attorney General’s Reference (No 1 of 2022)* [2022] EWCA 1259, [2023] 2 WLR 651. Subsequently, the Supreme Court (*Reference by the Attorney General for Northern Ireland—Abortion Services (Safe Access Zones) (Northern Ireland) Bill* [2022] UKSC 32, [2023] AC 505) has similarly ruled to restrict the reach of *Ziegler* (n 89).

91 We observed the appeal hearing.

92 Three interviews before the trial, five afterwards (one defendant was interviewed twice).

93 And formally unattributed.

94 We discuss these methods in greater detail elsewhere: Hayes, Cammiss and Doherty (n 77).

95 BSA, *Statement of Ethical Practice* (British Sociological Association 2017) <www.britsoc.co.uk/media/24310/bsa_statement_of_ethical_practice.pdf> accessed 6 August 2023.

96
*Roberts* (n 72) [26], [27] and [30].

97 ibid [26].

98 ibid [30].

99 ibid [26].

100 Above (n 21).

101 Submission to the Court of Appeal on behalf of Liberty (n 73).

102 Fieldnotes (n 74).

103 Above (n 21).

104
*Roberts* (n 72) [35].

105 ibid [50].

106 ibid [50].

107 ibid [49].

108 Statement by Ram at Basildon police station, 29 March 2017. Eleven of the defendants used all of the phrases ‘not lawful’, ‘risk of death or serious injury’, ‘genuinely and reasonably believed’ and ‘in a reasonable and proportionate way’, or close derivations thereof, in their short statements. These statement were included in the Agreed Facts of the case.

109 Fieldnotes, Chelmsford Crown Court, 6 and 8 November 2018. Clayson was understandably often hesitant in cross-examination, though this phrase was delivered fluently, like a prepared line.

110 Fieldnotes, Chelmsford Crown Court, 8 November 2018.

111 In its briefing prior to the original trial, End Deportations describes deportation flights as ‘brutal, secretive and barely legal’; the Home Office’s practice of racial profiling to fill seats on flights as ‘racist and arbitrary’; and the detention system as ‘inhumane’. End Deportations Media Briefing, March 2018. In contrast, the line developed by Ben Smoke in cross-examination—‘I don’t believe that our deportation system is fit for purpose. I would like a better and more liberal system’—is strikingly less oppositional (Fieldnotes, Chelmsford Crown Court, 6 November 2018). The defence was denied their request to call Home Office witnesses to explore the nature of its deportation decision making.

112 Fieldnotes, Chelmsford Crown Court, 29 November 2018.

113 ibid.

114 <https://detainedvoices.com/2017/03/27/my-ex-husband-said-he-knows-i-am-being-deported-next-week-he-is-waiting-for-me-he-is-planning-to-kill-me/> accessed 6 August 2023.

115 Fieldnotes (n 112).

116 ibid.

117 Fieldnotes, Chelmsford Crown Court, 9 and 13 November 2018.

118 Interview, January 2019.

119 Interview, February 2019.

120 Fieldnotes, Chelmsford Crown Court, 16 October 2018.

121 Fieldnotes, Chelmsford Crown Court, 21 November 2018.

122 Fieldnotes, Chelmsford Crown Court, 22 November 2018.

123
*Thacker* (n 52). For commentary, see Cammiss, Hayes and Doherty (n 25).

124 Fieldnotes, Bristol Crown Court, 13 December 2021.

125 Fieldnotes, Bristol Crown Court, 4 January 2022.

126 The SMV was initially constituted as an association of maritime merchants, but now acts as a third sector organisation. Historically, it has administered the charitable bequests made by Colston.

127 Fieldnotes, Bristol Crown Court, 15 December 2021.

128 ibid.

129 Fieldnotes, Bristol Crown Court, 16 December 2021.

130 Fieldnotes, Bristol Crown Court, 20 December 2021.

131 Fieldnotes (n 129).

132 ibid.

133 ibid.

134 ibid.

135 Fieldnotes (n 130).

136 Fieldnotes (n 127).

137 Fieldnotes, Bristol Crown Court, 22 December 2021.

138 Fieldnotes (n 129).

139 Fieldnotes (n 130).

140 Fieldnotes (n 129).

141 Fieldnotes, Bristol Crown Court, 14 December 2021.

142 Fieldnotes (n 137).

143 ibid.

144 ibid.

145 Tom Wainwright, Fieldnotes (n 125).

146 ibid.

147 ibid.

148 ibid.

149 Liam Walker, Fieldnotes (n 125).

150 Fieldnotes (n 129).

151 Fieldnotes (n 137).

152 ibid.

153 Fieldnotes (n 124).

154 Graham, Fieldnotes (n 130).

155 ibid.

156 ibid.

157 ibid.

158 Liam Walker, Fieldnotes (n 125).

159 Fieldnotes (n 130).

160 Goffman (n 4) 139.

161 Antony Duff and others, ‘Introduction: Judgment and Calling to Account’ in Antony Duff and others (eds), *The Trial on Trial Volume Two: Judgment and Calling to Account* (Hart Publishing 2006) 7.

162 Duff (n 3) 45 and 46.

163 Above (n 21).

164 Fieldnotes (n 137).

165 Above (n 21).

166 ibid.

167
*Attorney General’s Reference (No 1 of 2022)* (n 90) [3].

